# PRRX1 upregulates PD-L1 in human mesenchymal stem cells

**DOI:** 10.1007/s11626-024-00911-5

**Published:** 2024-04-25

**Authors:** Taro Osawa, Daisuke Yamada, Tomoka Takao, Lu Ming, Takeshi Takarada

**Affiliations:** Department of Regenerative Science, Okayama University Graduate School of Medicine, Dentistry and Pharmaceutical Sciences, 2-5-1 Shikata-cho, Kita-ku, Okayama, 700-8558 Japan

**Keywords:** Mesenchymal stem cells, Immunomodulation, Paired-related homeobox 1, Programmed death-ligand 1

## Abstract

Mesenchymal stem cells (MSCs) have been demonstrated to be efficacious in clinical applications for the amelioration of immune disorders, including graft-versus-host disease (GvHD) and Crohn's disease. The immunosuppressive role of Programmed death-ligand 1 (PD-L1) in MSCs is pivotal, yet the regulatory mechanisms governing its expression remain to be fully elucidated. In this study, we explored the influence of paired-related homeobox (PRRX1), a determinant of multipotency and self-renewal in MSCs, on the expression of various surface antigens, notably PD-L1. Multiple isoforms of PRRX1 were found to augment the mRNA levels of MSC markers, such as *CD26* and *CD317*, with all isoforms elevating PD-L1 expression at both mRNA and protein levels. This study reveals that PRRX1 may act as a potential immunomodulatory factor in MSCs by regulating the PD-L1 pathway.

**Supplementary Information:**

The online version contains supplementary material available at 10.1007/s11626-024-00911-5.

## Introduction

Mesenchymal stem cells (MSCs), also known as mesenchymal stromal cells, are multipotent adult stem cells of non-hematopoietic origin derived from the mesoderm. They are present in various tissues, including but not limited to, the bone marrow, adipose tissue, skeletal muscle, dermis, placenta, liver, spleen, and thymus (Liu *et al*. 2009). MSCs are endowed with self-renewal capabilities and the potential to differentiate into audiogenic, chondrogenic, and osteogenic lineages (Pittenger *et al*. 1999). Beyond their isolation and differentiation capacities, MSCs have demonstrated therapeutic efficacy in the treatment of graft-versus-host disease (GvHD) in patients with severe steroid-resistant conditions (Ringdén *et al*. 2006). Additionally, MSCs have been investigated in various clinical trials for their effectiveness in treating autoimmune disorders in humans, such as Crohn’s disease (Ibraheim *et al*. 2018), Systemic lupus erythematosus (SLE) (Wang *et al*. 2014), and Rheumatoid arthritis (RA) (Park *et al*. 2018). Despite these advancements, the precise mechanisms by which MSCs modulate the immune system remain to be fully delineated.

Previous research has shown that mesenchymal stem cells (MSCs) express their immunosuppressive functions both in vitro and in vivo through the modulation of T cells, B cells, dendritic cells, and natural killer cells (Han *et al*. 2012; de Castro *et al*. 2019). The interaction between MSCs and the immune effector cells is characterized by a complex, multifaceted, and overlapping mechanism that encompasses both direct cells contact and the release of soluble mediators (Augello *et al*. 2005). Programmed death-ligand 1 (PD-L1), also referred to as B7-H1 and CD274, is postulated to be present on various cell types including non-hematopoietic cells, antigen-presenting cells, T cells, B cells, dendritic cells (DCs), macrophages, and mesenchymal stem cells (Sharpe *et al*. 2007). The engagement of PD-L1 with programmed death 1 (PD-1) on activated T cells is crucial for the modulation of immune responses (Jin *et al*. 2010). Moreover, it has been previously established that MSCs express PD-L1, which leads to the inhibition of T cell proliferation (Davies *et al*. 2017). The PD-L1-PD-1 pathway's importance has gained recognition in autoimmune diseases (Jin *et al*. 2010; Pedoeem *et al*. 2014) with studies showing that the PD-1 pathway can attenuate T cell activation and confer protection against autoimmune pathologies, as observed in GvHD (Cassady *et al*. 2018), type I diabetes (Falcone and Fousteri 2020) and SLE (Liao *et al*. 2017). Hence, a deeper exploration into the potential interplay between MSCs and the PD-L1/PD-1 pathway is imperative to elucidate the mechanisms underpinning MSC-based therapeutic strategies.

The paired-related homeobox (PRRX1) protein is a pivotal transcription factor during embryogenesis and serves as a distinctive marker for mesenchymal stem cells (MSCs) in adult bone marrow (Miwa and Era 2018). Previous investigations have posited that PRRX1 modulates the differentiation pathways of multipotent mesenchymal progenitors. Illustratively, PRRX1-positive cells have been implicated in promoting pituitary organogenesis (Higuchi *et al*. 2014; Shintani and Higuchi 2021), osteogenesis, and chondrogenesis (Kawanami *et al*. 2009; Miwa and Era 2018). Overexpression of PRRX1 has been associated with a significant enhancement of regenerative processes in an aged mouse bone defect model (Xiao *et al*. 2020). Moreover, the upregulation of PRRX1 has proven efficacious in differentiating brown adipose-derived stem cells into sinus node-like cells (Yin *et al*. 2019). Additionally, PRRX1 has been shown to be crucial for the preservation of self-renewal capacities in adult neural stem cells (Shimozaki *et al*. 2013). Selective splicing of PRRX1 also generates isoforms with different C-termini, PRRX1A, PRRX1B and PRRX1C. PRRX1A retains the OAR domain, but other isoforms have not been identified (Norris and Kern 2001). The various PRRX1 variants are identical from the N-terminus to 167 amino acids, including the homeobox domain. Several papers demonstrate the functional differences of each PRRX1 isoform (Norris and Kern 2001; Takano *et al*. 2016; Li *et al*. 2017; Marchand *et al*. 2019), but their roles in BM-hMSC have not been defined. Nonetheless, the specific role of PRRX1 in MSCs remains enigmatic.

In this study, we explored the association between PRRX1 and a spectrum of surface molecules, including PD-L1, in bone marrow-derived human MSCs (BM-hMSCs). Our findings reveal that PRRX1 positively modulates the expression of PD-L1 in BM-hMSCs, suggesting a potential immunoregulatory function inherent in BM-hMSCs.

## Materials and methods

### Cell lines and cell culture

Bone marrow-derived human mesenchymal stem cells (BM-hMSCs) were procured from LONZA (Catalog #0,000,494,678) and cultured in Minimum Essential Medium Alpha (MEMα; Fuji-Film Wako, Japan) enriched with 20% fetal bovine serum (FBS; Hyclone, Victoria, Australia). The LentiX293T cell line was acquired from Takara Bio (Kusatsu, Shiga, Japan) and maintained in Dulbecco’s Modified Eagle Medium (DMEM; Fuji-Film Wako, Japan) supplemented with 10% FBS, penicillin (100 U/mL), and streptomycin (100 mg/mL) (Thermo Fisher Scientific, Waltham, MA). All cell lines were incubated in a humidified chamber with 5% CO2 at 37°C.

### RNA extraction and qRT-PCR

Total RNA was extracted utilizing ISOGEN reagent (Nippon Gene, Japan), followed by cDNA synthesis employing M-MLV Reverse Transcriptase (Thermo Fisher Scientific) and oligo-dT primers (Sigma-Aldrich, St. Louis, MO). The expression profiles of targeted genes were quantitatively assessed via qRT-PCR using the AriaMX Real-Time PCR System (Agilent Technologies, Santa Clara, CA). The amplification protocol entailed 40 cycles, consisting of a denaturation step at 95°C for 30 s, annealing at 62°C for 30 s, and extension at 72°C for 30 s. Primer sequences are delineated in Supplementary Table 1.

### Construction of each PRRX1 isoform-specific lentiviral vectors

Each PRRX1 isoform was amplified from BM-hMSC-derived cDNA utilizing KOD-FXneo (TOYOBO, New York, NY) with specific primers which sequences are delineated in Supplementary Table 1.

The lentiviral vector plasmid CSII-CMV-MSC, acquired from RIKEN BioResource Research Center (RDB04377, Kyoto, Japan), was subjected to restriction digestion using XbaI and EcoRI enzymes. The resultant digested vector was then ligated with the amplified PRRX1 isoforms employing Ligation High Ver 2 (Takara Bio).

### Production and infection of lentivirus

Lentiviral vectors were generated by co-transfecting lentiX293T cells with CSII-CMV-MSC constructs and the requisite packaging vectors—pMDLg/pRRE, pRSV-Rev, and pMD2.G—employing PEI-MAX transfection reagent (Polysciences, Warrington, PA, USA). At 12 h post-transfection, the culture media were refreshed. Subsequently, 48 h post-transfection, the lentiviral-containing culture supernatants were filtered through a 0.45 μm PVDF membrane (Hawach Scientific, Xi’an, China). The harvested lentiviral preparations were preserved at −80°C for subsequent applications. For viral transduction, MSCs were incubated with the lentiviral solution for 24 h. Post 4 days of cultivation, lentivirus infected MSCs were harvested for downstream experiments.

### Western blotting

Total protein was isolated using a lysis buffer composed of 0.1 M Tris (pH 6.7) and 4% SDS. Protein concentrations were determined by the bicinchoninic acid (BCA) protein assay kit (Thermo Fisher Scientific), with absorbance measured at 450 nm using the Multiskan Sky Microplate Spectrophotometer (Thermo Fisher Scientific). For electrophoresis, 10 μg of protein samples were resolved by SDS-PAGE and subsequently electrotransferred onto 0.45 μm PVDF membranes (Millipore, Burlington, MA). These membranes were then blocked with 5% (w/v) skim milk in 0.02% (v/v) Tween 20/PBS solution. Primary antibodies were applied at a 1:2000 dilution and incubated overnight at 4°C. This was followed by treatment with HRP-conjugated secondary antibodies at a 1:5000 dilution (GE Healthcare, Chicago, IL). Signal detection was achieved using Immobilon ECL Ultra Western HRP Substrate (WBULS0100, Merck, Billerica, MA) and visualized on an Amersham Imager 600 (Amersham). The antibodies utilized included PRRX1 (Novusbio, NBP2-13,816/Sigma-Aldrich, HPA051084), and HRP-conjugated anti-rabbit IgG (#7074, Cell Signaling Technology, Danvers, MA).

### Flow cytometry

The cells were resuspended in 100 μL of PBS supplemented with 2% FBS and phycoerythrin-conjugated human CD274 (PD-L1) antibody (PE-hCD274; #329,705, Biolegend, San Diego, CA) at a 1:200 dilution. This suspension was incubated on ice for 1 h. After incubation, the cells were washed with 2% FBS/PBS, and the fluorescence emitted by PE-hCD274 was measured. Detection and analysis were performed using a CytoFLEX S flow cytometer (Beckman Coulter, Brea, CA) and FlowJo software (FlowJo LLC, Ashland, OR), respectively.

### Statistical analysis

Data analysis was executed utilizing GraphPad Prism version 8 (GraphPad Software, San Diego, CA). All datasets represent biological replicates from three independent experiments and are expressed as means ± SEM. Statistical significance was assessed employing a two-tailed t-test and unpaired one-way or two-way ANOVA, followed by Tukey’s multiple comparisons test as post hoc analysis.

## Results

### Upregulation of MSC markers by PRRX1

**Figure 1.**
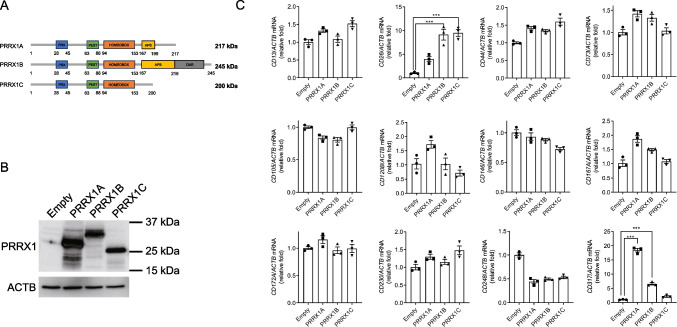
Transfection of PRRX1 and expression of genes associated with MSCs. (*A*) Information about each PRRX1 isoform. (*B*) Detection of PRRX1 by western blot analysis in PRRX1 overexpressed MSCs. Total protein was extracted from MSCs infected with lentivirus expressing the vector alone or PRRX1A, PRRX1B, PRRX1C, respectively. (*C*) RT-PCR analysis of surface markers in MSCs after PRRX1 overexpression. MSCs were infected with each PRRX1 isoform as reference and total RNA was purified to compare the expression level of genes associated with MSCs. All values were normalized to *ACTB* mRNA level (*n* = 3).

To elucidate the impact of the three PRRX1 isoforms on BM-hMSCs, we engineered lentiviral vectors to express PRRX1A, PRRX1B, and PRRX1C (Fig. 1*A*). Following viral transduction, total protein was isolated, and the presence of each isoform was verified by Western blot analysis (Fig. 1*B*). Comparative quantification of MSC markers, specifically CD13, CD26, CD44, CD73, CD105, CD120B, CD146, CD167, CD172A, CD230, CD248, and CD317, was performed via qRT-PCR. Notably, CD26 and CD317 expression levels were significantly elevated in the presence of PRRX1B or PRRX1C and PRRX1A or PRRX1B, respectively (Fig. 1*C*). These findings indicate that PRRX1 may play a modulatory role in the expression of a subset of MSC markers.

### Upregulation of PD-L1 in BM-hMSCs by PRRX1

**Figure 2.**
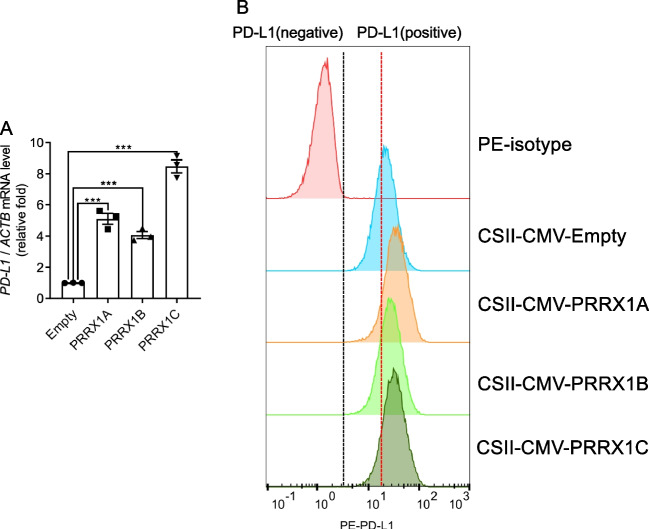
Upregulation of PD-L1 by PRRX1 Isoforms in BM-hMSCs. (*A*) qPCR analysis comparing PD-L1 mRNA expression levels. Following lentiviral transduction, total RNA was extracted from BM-hMSCs and the PD-L1 mRNA expression was quantified. Expression levels were normalized to ACTB mRNA (*n* = 3, representing three independent biological replicates). (*B*) Flow cytometric analysis of PD-L1 surface expression post-transduction with each PRRX1 isoform. The surface levels of PD-L1 on BM-hMSCs were assessed after infection with the respective lentiviral vectors for PRRX1 isoforms.

To assess the modulatory effect of PRRX1 on PD-L1, we measured the mRNA expression levels of PD-L1 after the expression of each PRRX1 isoform. Remarkably, all PRRX1 isoforms significantly increased the mRNA levels of PD-L1 (Fig. 2*A*). Furthermore, flow cytometric analysis demonstrated that the cell surface expression of PD-L1 was augmented by all PRRX1 isoforms as well (Fig. 2*B*). Collectively, these observations suggest that PRRX1 acts as a positive regulator of PD-L1 expression in BM-hMSCs.

## Discussion

Mesenchymal stem cells (MSCs) are heralded as one of the most promising cellular therapies due to their inherent self-renewal capability and potential to differentiate into diverse cell lineages. Furthermore, MSCs are recognized for their immunomodulatory functions and have been utilized in treating conditions such as graft-versus-host disease (GvHD) and autoimmune disorders, including Crohn’s disease (Naji *et al*. 2019). Despite advancements in MSC-based immunotherapy, its clinical application remains in nascent stages.

MSCs modulate the immune system through altering the functions of T cells, B cells, dendritic cells, and natural killer cells, primarily through direct cell interactions and the paracrine release of cytokines, growth factors, and chemokines such as transforming growth factor-β1 (TGF-β1), tumor necrosis factor-α (TNF-α), prostaglandin E2 (PGE2), interferon-γ (IFN-γ), and indoleamine 2,3-dioxygenase (IDO) (Song *et al*. 2020). Notably, IFN-γ is considered crucial for MSCs' immunosuppressive activity, as it can elevate PD-L1 expression on MSC surfaces, thereby significantly inhibiting T cell proliferation (Sheng *et al*. 2008). We are on the way to test the effect of IFN-γ or other PD-L1-inducible cytokines on PRRX1 expression in our future studies. Additionally, Davies's group has also proposed that MSCs secrete PD-1 ligands, suppressing activated T cell activity (Davies *et al*. 2017). Therefore, delineating the influence of MSCs on the PD-L1 pathway is essential for a more comprehensive understanding of their role in immunotherapy. In this vein, our study seeks to examine alterations in PD-L1 expression on MSCs to elucidate a potential mechanism within the PD-L1 pathway. Furthermore, we aim to explore the role of MSCs in autoimmune diseases and transplant rejection by examining PD-L1 expression changes using appropriate animal models. We also plan to assess the therapeutic potential and safety of these cells across a range of conditions, including cancer. This will allow us to evaluate the immunosuppressive effects attributable to the biological properties of PD-L1-overexpressing MSCs.

Preliminary studies have established that PRRX1 is critical in facilitating differentiation and sustaining the self-renewal capacity of MSCs (Shimozaki *et al*. 2013). However, literature on the influence of PRRX1 on the immunomodulatory functions of MSCs remains sparse. In this context, our research posits that PRRX1 may alter the immunoregulatory attributes of MSCs. Consequently, we constructed isoform-specific lentiviral vectors for PRRX1 to investigate this hypothesis by inducing overexpression in MSCs. Our results show a marked upregulation of PD-L1 on the cellular surface following PRRX1 overexpression. In addition, each PRRX1 isoform showed different regulatory effects on several MSC marker genes including CD26 and CD317 (Fig. 1*C*). Although the C-terminal sequence of each PRRX1 isoform is different (Fig. 1*A*) and their functional comparison of PRRX1 isoforms have been demonstrated by several groups (Norris and Kern 2001; Takano *et al*. 2016; Li *et al*. 2017; Marchand *et al*. 2019), the future study should reveal the detail regulatory mechanism. Furthermore, variations in the C-terminal sequence suggest that this part of the gene may be involved in the expression of each gene. Additionally, PRRX1 overexpression enhanced the expression of other critical MSC surface markers, such as CD44 and CD73 (Ramos *et al*. 2016). These insights suggest that PRRX1 could be a significant determinant in the regulation of MSC immunomodulatory functions via the PD-L1 pathway. Although Wnt/β-catenin signaling upregulates PRRX1 during human pluripotent stem cell-limb bud mesenchymal cell induction (Yamada *et al*. 2021), further studies are needed to test the effect of Wnt/β-catenin signaling on PRRX1-mediated PD-L1 expression.

In addition, PRRX1 inhibitors are currently not available, but the inhibitory effect of PRRX1 knockdown or knockout on PD-L1 expression should be tested in future studies.

Immunotherapy has demonstrated efficacy, to a degree, in haematological malignancies such as leukaemia and lymphoma, and in certain cases, neuroblastoma (Yu *et al*. 2010; Maude *et al*. 2014; Ladenstein *et al*. 2020). However, its therapeutic success in solid tumours remains suboptimal (Ai *et al*. 2020). For instance, paediatric sarcomas may induce immunosuppressive pathways within the tumour microenvironment (TME), thereby diminishing the effectiveness of immunotherapy (Dyson *et al*. 2019).There have been limited clinical trials involving immunotherapies targeting PD-1 and other immune checkpoints for Ewing's sarcoma (Morales *et al*. 2020; Clemente *et al*. 2021). Moreover, the TME may impair the activity of CD8 + T cells, which are expected to be primed by macrophages to target tumour cells, thus mitigating their cytotoxic function, and potentially leading to rapid exhaustion via the PD1/PDL1 interaction (Ai *et al*. 2020). Conversely, MSCs are posited to counteract tumour suppression by repopulating macrophages within the TME, exerting immunomodulation, inhibiting tumour progression, and preventing autoimmune rejection (Aggarwal and Pittenger 2005). Nonetheless, the extent to which PRRX1 exerts comparable effects in MSCs derived from sources other than bone marrow is uncertain, as MSCs from varied tissues and species may exhibit distinct biological characteristics (Uder *et al*. 2018). Consequently, additional research is imperative to address these uncertainties.

## Conclusions

**Figure 3.**
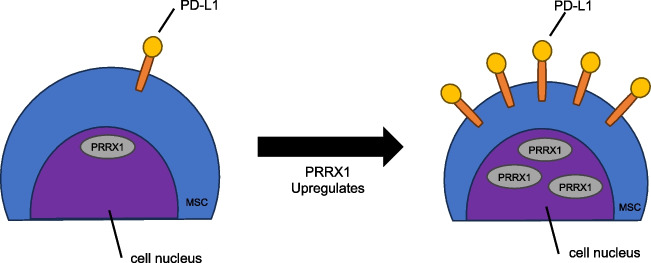
Schematic model of PRRX1 overexpression was significantly associated with PD-L1 upregulation in MSCs.

This study is the inaugural report demonstrating that the overexpression of PRRX1 elevates PD-L1 expression and enhances the expression of other MSC surface markers (Fig. 3). These findings implicate PRRX1 as a potentially pivotal element in the immunoregulatory network of MSCs. Moreover, PRRX1 is anticipated to emerge as a novel therapeutic target for modulating the PD-L1 pathway in MSCs.

## Supplementary Information

Below is the link to the electronic supplementary material.

Supplementary file1 (PDF 18 kb)
