# Correction: PRRX1 upregulates PD-L1 in human mesenchymal stem cells

**DOI:** 10.1007/s11626-026-01202-x

**Published:** 2026-07-27

**Authors:** Taro Osawa, Daisuke Yamada, Tomoka Takao, Lu Ming, Takeshi Takarada

**Affiliations:** Department of Regenerative Science, Okayama University Graduate School of Medicine, Dentistry and Pharmaceutical Sciences, 2-5-1 Shikata-cho, Kita-ku, Okayama, 700-8558 Japan

**Correction: In Vitro Cellular & Developmental Biology - Animal (2024) 60:1132-1137**

**https://doi.org/10.1007/s11626-024-00911-5**

The name of coauthor Tomoka Takao was misspelled in the article as originally published.

The original article has been corrected.

