# Supplementary material for: PRRX1 upregulates PD-L1 in human mesenchymal stem cells
Source: In Vitro Cell Dev Biol Anim. 2024 Apr 25;60(10):1132–7. doi: 10.1007/s11626-024-00911-5 (PMC11655573; doi:10.1007/s11626-024-00911-5)
Supplement: Supplementary file 1 — Supplementary file1 (PDF 18 kb) [file 11626_2024_911_MOESM1_ESM.pdf]

Supplementary Table.1

qRT-PCR primers

Gene	Forward primer sequence	Reverse primer sequence
ACTB	AGAAAATCTGGCACCACACC	AGAGGCGTACAGGGATAGCA
CD13	GACCAAAGTAAAGCGTGGAATCG	TCTCAGCGTCACCCGGTAG
CD26	GGGTCACATGGTCACCAGTG	TCTGTGTCGTTAAATTGGGCATA
CD44	CTGCCGCTTTGCAGGTGTA	CATTGTGGGCAAGGTGCTATT
CD73	GCCTGGGAGCTTACGATTTTG	TAGTGCCCTGGTACTGGTCG
CD105	TGCACTTGGCCTACAATTCCA	AGCTGCCCACTCAAGGATCT
CD120B	CGGGCCAACATGCAAAAGTC	CAGATGCGGTTCTGTTCCC
CD146	AGCTCCGCGTCTACAAAGC	CTACACAGGTAGCGACCTCC
CD167A	AAGGGACATTTTGATCCTGCC	CCTTGGGAAACACCGACCC
CD172A	GGCCTCAACCGTTACAGAGAA	GTTCCGTTCATTAGATCCAGTGT
CD230	AGTCAGTGGAACAAGCCGAG	CTGCCGAAATGTATGATGGGC
CD248	TGGTGCCAACGTGTGTCTTTT	AGCGATAGCAGTCAGTGATGC
CD317	CACACTGTGATGGCCCTAATG	GTCCGCGATTCTCACGCTT
PD-L1	AAATGGAACCTGGCGAAAGCgc	GATGAGCCCCTCAGGCATTT

cloning primer

Gene	Forward primer sequence	Reverse primer sequence
PRRX1A	GAATTCgccaccatgacctccagctacgggcac	TCTAGAttagaatccgttatgaagcc
PRRX1B	GAATTCgccaccatgacctccagctacgggcac	TCTAGAtcagttgactgttggcacc
PRRX1C	GAATTCgccaccatgacctccagctacgggcac	TCTAGATCACCTGTACGGAGACGCTG.
